# *Neorickettsia* sp. in coatis (*Nasua nasua*) in Brazil

**DOI:** 10.1590/S1984-29612023042

**Published:** 2023-07-17

**Authors:** Lívia Perles, Wanessa Teixeira Gomes Barreto, Gabriel Carvalho de Macedo, Heitor Miraglia Herrera, Rosangela Zacarias Machado, Marcos Rogério André

**Affiliations:** 1 Laboratório de Bioagentes Transmitidos por Vetores, Departamento de Patologia, Reprodução e Saúde Única, Faculdade de Ciências Agrárias e Veterinárias – FCAV, Universidade Estadual Paulista “Júlio de Mesquita Filho” – UNESP, Jaboticabal, SP, Brasil; 2 Programa de Pós-graduação em Ecologia e Conservação, Universidade Federal de Mato Grosso do Sul – UFMS, Campo Grande, MS, Brasil; 3 Laboratório de Biologia Parasitária, Ciências Ambientais e Sustentabilidade Agropecuária, Universidade Católica Dom Bosco – UCDB, Campo Grande, MS, Brasil

**Keywords:** Anaplasmataceae, procyonidae, Central-Western Brazil, Anaplasmataceae, procionídeo, Centro-Oeste brasileiro

## Abstract

The genus *Neorickettsia* comprises trematode-associated bacteria that can cause diseases in animals and humans. Despite detection of *Neorickettsia* antigens in the intestine of coatis kept in captivity in southern Brazil through immunohistochemistry, the molecular identity of the bacteria in South American procyonids remains elusive. The aim of the present study was to investigate the occurrence of *Neorickettsia* sp. in blood samples from coatis in central-western Brazil. Between March 2018 and January 2019, animals were captured and recaptured in two areas of the Cerrado (*Parque Estadual do Prosa*, PEP; and *Vila da Base Aérea*, VBA) located in the city of Campo Grande, state of Mato Grosso do Sul, central-western Brazil. All captures were performed according to convenience. DNA from 97 blood samples was subjected to nested PCR (nPCR) targeting a fragment of the 16S rRNA gene of *Neorickettsia* sp. Six samples (3.6%; five from VBA and one from PEP) from different coatis were positive in nPCR based on the 16S rRNA. The sequences obtained (~500 bp) showed ˃ 99% similarity to *N. risticii*. Phylogenetic analysis clustered the sequences detected in the present study in a clade with *N. risticii*. This is the first molecular detection of *Neorickettsia* sp. in coatis in Brazil.

## Resumo

O gênero *Neorickettsia* compreende bactérias associadas a trematódeos que podem causar doenças em animais e humanos. Apesar da detecção de antígenos de *Neorickettsia* por imuno-histoquímica no intestino de quatis mantidos em cativeiro no sul do Brasil, a identidade molecular da bactéria em procionídeos da América do Sul permanece indefinida. O presente trabalho teve como objetivo investigar a ocorrência de *Neorickettsia* sp. em amostras de sangue de quatis do Centro-Oeste do Brasil. Entre março de 2018 e janeiro de 2019, os animais foram capturados em duas áreas de Cerrado (Parque Estadual do Prosa PEP e Vila da Base Aérea VBA) localizadas na cidade de Campo Grande, Mato Grosso do Sul, Centro-Oeste do Brasil. Todas as capturas e recapturas foram realizadas por conveniência. O DNA de 97 amostras de sangue foi submetido a "nested" (nPCR), baseada em um fragmento do gene 16S rRNA de *Neorickettsia* sp. Seis (3,6% - 5 de VBA e 1 de PEP) amostras de quatis diferentes foram positivas na nPCR, baseada no rRNA 16S. As sequências obtidas (~500 pb) apresentaram ˃99% de identidade com *N. risticii*. A inferência filogenética agrupou as sequencias detectadas no presente estudo em um clado com *N. risticii*. Esta é a primeira detecção molecular de *Neorickettsia* sp. em quatis do Brasil.

Unlike other agents in the family Anaplasmataceae (Order Rickettsiales), which are transmitted by blood-sucking arthropods, the genus *Neorickettsia* comprises trematode-associated bacteria that can cause diseases in animals and humans (Dumler et al., 2001). Although several molecular studies have reported the presence of *Neorickettsia* DNA in different hosts, only four species have been fully characterized, given the great difficulty in cultivating and sequencing the complete genome of these bacteria. These species are *Neorickettsia sennetsu*, which causes Sennetsu neorickettsiosis in humans (Dittrich et al., 2015); *Neorickettsia helminthoeca*, which causes salmon poisoning disease (SPD) in dogs (Headley et al., 2011); and *Neorickettsia risticii* and *Neorickettsia findlayensis*, which are the etiological agents of Potomac horse fever (PHF) (Teymournejad et al., 2020).

In Brazil, *N. risticii* has already been molecularly detected in horses presenting clinical signs resembling Potomac fever in the states of Rio Grande do Sul (Coimbra et al., 2006) and Rio de Janeiro (Paulino et al., 2020). *N. risticii* DNA has also been detected in the snails *Heleobia piscium*, *Heleobia parchappei* and *Heleobia davisi* in the state of Rio Grande do Sul, where an outbreak of PHF had been previously reported (Coimbra et al., 2006). Parapleurolophocercous cercariae of trematodes identified in the snail *H. piscium* have also been found to contain *N. risticii* DNA (Coimbra et al., 2006). Recently, *Neorickettsia* sp. DNA was detected in 57 (13.63%) out of 418 biological samples from fruit bats in a periurban area of the city of Campo Grande, state of Mato Grosso do Sul, central-western Brazil (Ikeda et al., 2021).

SPD caused by *N. helminthoeca* is considered to be an endemic disease in dogs in the western parts of the USA and Canada (Headley et al., 2011). In Brazil, Headley et al. (2006) found anatomopathological lesions in 10 dogs in the city of Maringá, state of Paraná, which were consistent with alterations associated with SPD. Based on PCR assays targeting the *rpoB* and *groEL* genes, the presence of *N. helminthoeca* DNA was detected in tissues from dogs with pathological lesions consistent with SPD (Headley et al., 2006). *Neorickettsia helminthoeca* antigens were detected in the intestine of three ring-tailed coatis kept in a conservationist breeding facility in Foz do Iguaçu, southern Brazil, through immunohistochemistry (Headley et al., 2018). Intestine and spleen fragments were positive in a PCR for *N. helminthoeca* based on the 16S rRNA gene, but due to the low quality of the amplicons obtained, no sequence was obtained (Headley et al., 2018). The aim of the present study was to investigate the occurrence of *Neorickettsia* sp. in blood samples from coatis in central-western Brazil.

Between March 2018 and January 2019, coatis (*Nasua nasua*) were sampled every three months on 10 consecutive days in two urban areas: a conservation unit in the Prosa State Park (Parque Estadual do Prosa, PEP) (-20.44987, -54.56529) and a residential area named ‘Vila da Base Aérea’ (VBA) (-20.47163, -54.65405), located in the city of Campo Grande, state of Mato Grosso do Sul, central-western Brazil. The animals were anesthetized with an association of Tiletamine hydrochloride and Zolazepam hydrochloride (Telazol, Zoetis^®^; 7 mg/kg, intramuscularly). To be able to identify any animals that were caught again, the animals sampled were labeled with numbered colored ear tags and underwent implantation of a microchip in their subcutaneous tissue. Their age was estimated using the method of Olifiers et al. (2010). In total, 97 different coatis were sampled (42 PEP and 55 VBA; 56 females and 41 males; and 70 adults and 27 subadults). Blood was sampled from the femoral vein using tubes containing ethylenediamine tetra-acetic acid (EDTA) (Perles et al., 2022).

DNA was extracted from blood samples using the Illustra Blood Mini Kit (GE Healthcare^®^, Chicago, IL, USA), in accordance with the manufacturer’s instructions. All the DNA samples were initially subjected to PCR targeting the mammal endogenous glyceraldehyde 3-phosphate dehydrogenase (*gapdh*) gene (Birkenheuer et al., 2006). Positive samples were then subjected to nested PCR (nPCR) targeting a fragment (approx. 500 bp) of the 16S rRNA gene of *Neorickettsia* sp. (Chae et al., 2003). Samples that were positive for the 16S rRNA were further subjected to PCR assays targeting the nearly full-length 16S rRNA (Kanter et al., 2000), *p51kDa* and *groEL* genes (Barlough et al., 1998; Gibson et al., 2011; Cicuttin et al., 2013).

**Figure 1:**
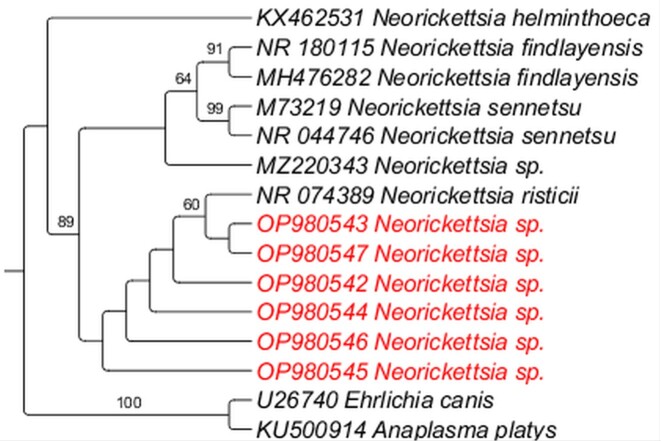
Phylogenetic tree based on Maximum likelihood inference of *Neorickettsia* spp. based on an alignment of 539 bp of the 16S rRNA gene. Sequences detected in the present study are highlighted in red.

All coatis’ blood DNA samples were positive in the PCR targeting the *gapdh* gene. Six (3.6%; five from VBA and one from PEP) different coatis were positive in the nPCR based on the 16S rRNA. The sequences obtained (~500 bp) showed ˃ 99% similarity (query coverage of 100% and E-value of 0.0) to *N. risticii*. Unfortunately, all of these six samples were negative in the additional PCR protocols targeting the *p51kDa*, *groEL* and nearly complete 16S rRNA genes, thus precluding robust phylogenetic inferences. The sequences generated and analyzed during the current study are available in the NCBI GenBank nucleotide platform (NCBI, 2022) and can be obtained through the following accession numbers: OP980542, OP980543, OP980544, OP980545, OP980546 and OP980547. For phylogenetic inferences, sequences from the present study were aligned with those retrieved from GenBank using MAFFT software version 7 (Katoh et al., 2019), with the best evolutionary model chose following the Akaike Information Criterion (AIC). Maximum likelihood (ML) phylogenetic analysis was performed using the iqTREE software (IQ-TREE, 2022) (Stöver & Müller, 2010). The phylogenetic tree edition and rooting (outgroup) were performed using TreeGraph 2.0 beta software. Maximum likelihood phylogenetic analysis clustered the sequences detected at the present study in a clade with *N. risticii* (Figure 1).

Although the sequences obtained here showed high similarity to *N. risticii*, Dumler et al. (2001) demonstrated phylogenetic heterogeneity among *N. risticii* 16S rRNA sequences. The previous study showed that mutations of up to 15 nucleotides can be present, with formation of different clades within the same species. In fact, a new *Neorickettsia* species has recently been described in an area where *N. risticii* was incriminated as the only etiological agent of PHF in horses (Teymournejad et al., 2020). Based on phylogenetic inferences from the nearly complete 16S rRNA, *p51kDa*, *Ssa3* and *Ssa1* genes, and on isolation and whole genome sequencing (WGS), Teymournejad et al. (2020) described *Neorickettsia findlayensis* in horses in Canada. Although the positive samples of the present study showed > 99% similarity to *N. risticii* samples that are available at GenBank, the real identity of the bacteria detected in ring-tailed coatis should be further investigated through isolation and WGS.

In the present study, *Neorickettsia* sp. was molecularly detected in blood samples from wild ring-tailed coatis in a periurban area in central-western Brazil. Since *Neorickettsia* sp. has now been molecularly detected in the same study area both in bats by Ikeda at al. (2021) and in wild coatis in the present study, we predict that these Anaplasmataceae agents might be maintained in periurban areas in central-western Brazil through an intricate transmission cycle involving free-living mammals. Further studies should be conducted with the aim of isolating the agents detected, in order to shed some light on the real identity of *Neorickettsia* circulating in wild animals in Brazil.
